# Editorial: Allogeneic transplantation in pediatric patients with hematologic malignancies

**DOI:** 10.3389/fped.2024.1411922

**Published:** 2024-04-26

**Authors:** Miguel Angel Diaz

**Affiliations:** Pediatric Hematology/Oncology Department, Niño Jesús University Children’s Hospital, Madrid, Spain

**Keywords:** allogeneic HSC transplantation, pediatric malignant diseases, caregivers perceptions, transplant outcomes, haploidentical donors

**Editorial on the Research Topic** Allogeneic transplantation in pediatric patients with hematologic malignancies

Allogeneic hematopoietic stem cell transplantation (HSCT) stands as the unique curative option for some pediatric patients with malignant mainly high-risk leukemias and non-malignant diseases such as sickle cell anemia, thalassemia, and severe combined immunodeficiency. However, and despite clinical outcomes have been improved over last two decades, caregiveŕs knowledge and perception about allogeneic HSCT can significantly influence the decision-making process surrounding HSCT for these young patients specially in cases of non-malignant diseases. This can negatively impact on a wider use of allogeneic HSCT in pediatric patients. Monagel et al., hypothesized that caregivers knowledge remains insufficient what leading a restriction on acceptation of allogeneic HSCT for sickle cell disease patients in Saudi Arabia. To address this hypothesis, they performed a cross-sectional study using a survey distributed by electronic devices to parents and/or caregiver patients. They found that around 50% of survey responders declared that the main reason for HSCT rejection was worry and fear about transplant side effects. Moreover, 36% of them declared misconception towards transplant procedure even lack of knowledge in 13% of survey responders. These findings highlight the great effort to be made in the future by health professionals. Caregivers cultural beliefs and deep-rooted cultural stigmas or misconceptions surrounding HSCT may deter families from pursuing this treatment option, despite its potential to alleviate the burden of disease and improve quality of life. Education and awareness initiatives must bridge this gap, dispelling myths and fostering informed decision-making grounded in scientific evidence rather than unfounded fears. In this regard, gain new information about long-term survival and quality of life of pediatric patients underwent allogeneic HSCT should help clinicians to better advise caregivers in the decision-making process. In this line, Molina et al., retrospectively analyze the long-term transplant outcomes and the variables that impact in this endpoint. They performed a landmark analysis in a group of 162 pediatric patients that received an allogeneic HSCT for malignant diseases. Landmark analysis studies on pediatric patients are especially important considering that a relevant proportion of them become lost to follow-up. Authors found that long-term survival is positively influenced by good immune reconstitution and negatively by the presence of severe chronic graft-vs.-host disease (GvHD). They concluded that presence of severe cGvHD is the most important factor that negatively impacts on transplant outcomes in patients who survived beyond 1 year after the transplant. Transplant strategies should aim to reduce the risk of such a devastating long-term complication. This connects with the important question of pediatric quality of life (QoL) following allogeneic HSCT and how to evaluate it. QoL can be impaired by long-term adverse events including organ dysfunction, secondary cancers and cGvHD. Despite this, QoL has been little studied in the pediatric transplant setting. QoL is usually assessed by objective and subjective parameters. The Pediatric Quality of Life Inventory (PedsQL) is authorized to asses QoL in children aged 2–18 years old. In this respect, Wei et al., performed a cross-sectional study assessing a Chinese mandarin version of PedsQL and its reliability. Authors concluded that the Chinese mandarin version of PedsQL is feasible, reliable and valid to asses QoL of Chinese children after HSCT. It is important to note that several items in the English version were modified to cross-cultural adaptation. This is essential for navigating the diverse cultural background and perspectives of patients and their families in decision-making about HSCT.

Last but not least, Moreno et al., addressed the role of haploidentical transplant as alternative to HLA matched donor transplant. The use of alternative donors such as haploidentical donors have expanded the use of allogeneic transplants to pediatric patients lacking a matched sibling donor. Biological parents share at least one haplotype with their children and all of them could be considered as potential donors. Even siblings and other relatives might be potential haploidentical donors, which contributes to almost all pediatric patients can have at least one available donor in a timely manner for allogeneic HSCT. Initially the use of haploidentical transplantation was associated with many clinical problems but the advent of T-cell depletion techniques and the use of post-transplant cyclophosphamide as GvHD prophylaxis have changed the haploidentical transplant landscape leading a rapid increase of its use worldwide over the last few years. As such, it is the allogeneic transplant modality that has the most growth in recent years not only in adults but also in children. However, there are few studies that compare transplant outcomes in between both groups. Authors compared outcomes of children with acute lymphoblastic leukemia undergoing HSCT in second complete remission (CR2) from haploidentical vs. HLA-matched donors. A prospective data registry was generated, which allowed them to analyze treatment results. In this study, they focused on the analysis and comparison of treatment outcomes of 76 children undergoing HSCT in CR2 from haploidentical vs. HLA-matched donors. They found no significant differences in the estimate of principal transplant endpoints between both groups. Despite the short follow-up period of study, these results support the role of haploidentical donors as an alternative to HLA- compatible donors in this population.

In conclusion, while the promise of HSCT offers a glimmer of hope for pediatric patients with malignant and non-malignant diseases, its realization hinges upon our collective commitment to addressing the social determinants that shape healthcare access and decision-making. Only through concerted efforts to dismantle barriers can we ensure that every child, regardless of background or circumstance, has the opportunity to embrace a future unburdened by disease.

